# Risk factors for developing intra-abdominal abscess following appendicectomy for acute appendicitis: a retrospective cohort study

**DOI:** 10.1007/s00423-024-03421-w

**Published:** 2024-08-09

**Authors:** B P Mao, G Collins, F E Ayeni, D J Vagg

**Affiliations:** 1 Department of Surgery, Nepean Hospital, Sydney, NSW Australia; 2 Faculty of Medicine and Health, The University of Sydney, Sydney, NSW Australia; 3 Nepean Institute of Academic Surgery, Nepean Clinical School, Sydney, NSW Australia; 4 Concord Repatriation General Hospital, Sydney, NSW Australia

## Abstract

**Background:**

Laparoscopic appendicectomy is commonly performed in Australia for treatment of acute appendicitis. Intra-abdominal abscess (IAA) is a potential complication following appendicectomy for acute appendicitis. Risk factors for developing post-operative IAA remain controversial and poorly defined. Laparoscopic washout may be performed for patients who develop complication(s) including IAA. The aim of this study was to define risk factors for both the development of IAA and identify patients who may require laparoscopic washout following appendicectomy.

**Methods:**

Data were obtained from 423 patients who underwent laparoscopic appendicectomy over a five-year period (2012–2017). Clinical (fever, haemodynamics, examination findings), biochemical (white cell count, neutrophil count, C-reactive protein, bilirubin, albumin), radiological (CT free fluid), and operative factors (inflammation, suppuration, free-fluid, perforation, histopathology) collected in the pre-, peri-, and post-operative period(s) were analysed.

**Results:**

23 (5.4%) patients developed post-operative IAA. Duration of intravenous antibiotics was significantly longer in patients who developed IAA and in those who required laparoscopic washout (*p* < 0.0001). C-reactive protein (CRP) on admission (*p* < 0.05) and appendiceal perforation (*p* = 0.0005) were significantly higher in patients who either developed IAA or needed laparoscopic washout. No clinical or radiological finding predicted either the development of IAA or need for laparoscopic washout.

**Conclusion:**

Elevated CRP on admission may predict the development of post-operative IAA formation or the need for laparoscopic washout post-appendicectomy. Prolonged post-operative antibiotic use appears independent of the development of IAA as well as the need for laparoscopic washout. These data highlight the need for clear guidelines on peri-operative antibiotic use following appendicectomy.

## Introduction

Acute appendicitis is a common abdominal emergency in Australia. The rate of acute appendicitis in developed countries is 90–100 patients per 100,000 inhabitants per year [1]. In Australia, laparoscopic appendicectomy has become the standard procedure for managing acute appendicitis in both adult and paediatric populations, with benefits of reduced post-operative pain, fewer overall complications, lower rates of wound infection, shorter hospital stay and decreased recovery time [2, 3]. Non-operative management of acute appendicitis has been shown to have similar efficacy and safety to surgical management for uncomplicated appendicitis, however it has been associated with longer hospital stay and higher rate of recurrence when compared to laparoscopic appendicectomy [4].

Post-operative intraabdominal abscess (IAA) is a potential complication following laparoscopic appendicectomy with an overall incidence of approximately 2.2% in the adult population [2]. Post-operative IAA is associated with significant morbidity, longer hospital stays and double the hospital costs [5]. Management of IAA includes antibiotic therapy with or without percutaneous drainage of the abscess, laparoscopic washout and potential re-operation. Prophylactic laparoscopic washout may also play a role in preventing development of IAA [6] as well as managing other complications arising from laparoscopic appendicectomy including a persistent systemic inflammatory response syndrome (SIRS), persistent abdominal pain and peritonism.

Risk factors for IAA following laparoscopic appendicectomy remain controversial and poorly defined. Those previously identified include body mass index (BMI) > 30, gangrene and perforation, pelvic peritonitis, peritoneal irrigation, operative time > 90 min and pre-operative leukocytosis > 20,000/mm^3^. Of these, perforated appendicitis is the only independent risk factor consistently identified [7–12]; according to summarised data from multiple studies, the risk of developing post-operative IAA in non-perforated appendicitis is 1% compared to 5–10% in perforated appendicitis [8].

The aim of this study was to further define clinical, radiological, biochemical and operative risk factors for both the development of post-operative IAA and for patients who may require laparoscopic washout following appendicectomy.

## Methods

This was a retrospective cohort study which included all patients who underwent laparoscopic appendicectomy for acute appendicitis during a five-year period (2012–2017) at Nepean Hospital, Sydney, Australia. Nepean Hospital is a large, tertiary referral hospital within the Nepean Blue Mountain Local Health District which services a population covering 9179km^2^. Data were collected from the Acute Surgical Unit (ASU) database which is prospectively maintained with updates made regularly throughout the day reflecting real-time changes in patient care. Follow up data were collected if the patient developed a complication during the admission or re-presented to the Emergency Department with a post-operative complication following laparoscopic appendicectomy.

Management of acute appendicitis was directed by locally developed hospital guidelines which are based on retrospective local data and in consultation with the infectious diseases department who advised on peri-operative antibiotic administration. All emergency surgical patients with a diagnosis of acute appendicitis over the age of five (5) were admitted and managed by the Acute Surgical Unit; patients younger than five years were transferred to a dedicated Children’s hospital for their management. All patients included in this study underwent laparoscopic appendicectomy during their admission once a diagnosis of acute appendicitis had been made.

The following data were collected: (1) demographics (age, sex, American Society of Anesthesiology (ASA) score and Charlson Comorbidity Index (CCI)); (2) clinical variables (vital signs (temperature, respiratory rate, heart rate); (3) symptoms and their duration (nausea, vomiting, anorexia, migratory pain, right iliac fossa (RIF) pain, peritonism). Biochemical parameters were measured both pre- and post-operatively. These included white cell count (WCC), neutrophil count, C-Reactive Protein (CRP), bilirubin and albumin. Vital signs, WCC, and CRP were measured on the day of surgery and post-operative days one, two and three if the patient was still admitted in hospital. Radiological findings (if performed) included computed tomography (CT) peri-appendiceal changes, peri-appendiceal collection, free fluid and free gas. Peri-operative data including operative findings, surgical approach, appendiceal perforation, complications, time from admission to first antibiotic dose, post-operative antibiotic duration, length of stay, and readmission rate were also obtained. All operative specimens were sent for formal histopathology and correlated to intra-operative findings.

Acute appendicitis was diagnosed using clinical findings, laboratory investigations and radiology (where indicated and/or performed). A CT of the abdomen and pelvis was performed in patients if an alternative diagnosis was felt to be equally or more likely. Some patients transferred to our hospital for management or sent in for admission from the community presented with pre-operative imaging already available. All patients received peri-operative antibiotics once a decision to proceed with surgery had been made. Unless contraindicated, broad spectrum antibiotics to cover gram negative, gram positive and anaerobic bacteria were administered. Laparoscopic washout was not routinely performed during the index laparoscopic appendicectomy.

Post-operative IAA was suspected in patients with post-operative fever, tachycardia, peritonism and/or persistent abdominal pain. In the immediate post-operative period clinical findings were used to make the provisional diagnosis and direct operative management; cross-sectional imaging was used only if an alternate diagnosis was thought to be more likely responsible for the patient’s clinical disposition. Patients with clinical suspicion of an IAA diagnosed within 48-hours post-appendicectomy underwent a laparoscopic washout, often confirming a diagnosis of IAA. If the patient represented to the outpatient clinic or emergency department with clinical suspicion for IAA, cross-sectional imaging was first performed to support the diagnosis prior to further management. Subsequent management consisted then of antibiotics with percutaneous drainage (if amenable to percutaneous access) or laparoscopic washout of the abdominal cavity after considering time since index appendicectomy.

Data was used to analyse three patient groups:

1. Post-operative IAA versus no post-operative IAA.
2. Post-operative IAA and laparoscopic washout versus no post-operative IAA and no laparoscopic washout.
3. Laparoscopic washout versus no laparoscopic washout.

Analysis was performed using GraphPad Prism software. Univariate descriptive analysis was performed on extracted data as required. Continuous variables were reported as a mean and range and categorical variables as a percentage. Differences between groups were measured using the Fisher’s exact test for categorical data and unpaired student t-test for continuous data. Significant level was accepted at *p* < 0.05.

Selection bias was minimised by including all patients who underwent laparoscopic appendicectomy during 2012–2017 with no other eligibility criteria. Information bias was minimised as the Acute Surgical Unit database is comprehensive and prospectively maintained. Due to limited sample size, patients with missing data were not excluded from the study.

## Results

Data were obtained from 423 patients. Demographic data and clinical variables are summarised in Table 1.

**Table 1:** Patient demographics and pre-, peri- and post-operative factors

**Patient demographics**	
Age (mean (±SD), *years*)	36.7 (±19.68)
Female (n (%))	177 (41.84)
Male (n (%))	246 (58.16)
ASA ≥ 2 (n (%))	180 (48.13)
CCI ≥ 3 (n (%))	37 (8.75)
**Pre-operative factors**	
Patients requiring CT (n (%))	206 (48.70)
CT peri-appendiceal change (n (%))	173 (83.98)
CT peri-appendiceal collection (n (%))	22 (10.67)
CT free fluid (n (%))	91 (44.17)
CT free gas (n (%))	33 (16.02)
CT faecolith (n (%))	83 (40.29)
Time from admission to surgery (mean (±SD), *hours*)	19.4 (±46)
**Peri-operative factors**	
Suppurative appendix (n (%))	30 (7.10)
Gangrenous appendix (n (%))	76 (17.97)
Perforated appendix (n (%))	269 (63.59)
Abscess (n (%))	89 (21.04)
**Post-operative factors**	
Histology: inflamed (n (%))	9 (2.13)
Histology: suppurative (n (%))	31 (7.33)
Histology: gangrenous and perforated (n (%))	383 (90.54)
Length of post-operative IV antibiotics (mean (±SD), *days*)	4.3 (±1.78)
Patients receiving post-operative oral antibiotics (n (%))	104 (24.59)
Length of post-operative oral antibiotics (mean (±SD), *days*)	6.2 (±2.69)
Post-operative IAA (n (%))	23 (5.44)
Laparoscopic washout (n (%))	18 (4.23)
Length of stay (mean (±SD), *days*)	5.3 (±2.22)
Readmission (n (%))	37 (8.75)

*ASA* American Society of Anesthesiologists; *CCI* Charlson Comorbidity Index; *CT* Computed tomography

The patient population was predominantly adult with mean age 36.7 years (SD ± 19.68). The youngest and oldest patient were aged 5 and 85 years respectively. There were 177 female patients (41.84%) and 246 male patients (58.16%). The ASA score was ≥ 2 in 48.13% of patients, correlating to mild systemic disease or above. The CCI was ≥ 3 in 8.75% of patients, correlating to moderate comorbid burden or above. Average time from hospital admission to surgery was 19.4 h (SD ± 46.00) and the average length of stay was 5.3 days (SD ± 2.22). There was an 8.75% readmission rate. Reasons for readmission included feeling generally unwell (2.78%), wound infection (13.89%), abdominal pain with (5.56%) or without fever (63.89%), abdominal pain and fever with outpatient CT finding of IAA (8.33%), vomiting and diarrhoea (5.56%).

Just under half of all patients underwent pre-operative cross-sectional imaging (*n* = 206; 48.70%); 173 (83.98%) had peri-appendiceal changes, 22 (10.67%) had a peri-appendiceal collection, 91 (44.17%) had free fluid, 33 (16.02%) had free gas and 83 (40.29%) had faecolith visible on imaging. Further data on pre-, peri- and post-operative clinical and biochemical risk factors are provided in the appendices.

Suppurative appendicitis was found during laparoscopic appendicectomy in 30 patients (7.10%) and gangrenous appendicitis in 76 patients (17.97%). Over half of patients had an appendiceal perforation (*n* = 269; 63.59%). Intra-abdominal abscess was encountered during laparoscopic appendicectomy in 89 patients (21.04%). 40 patients (9.46%) underwent conversion from laparoscopic appendicectomy to open procedure however analysis did not show any significant difference between the groups.

Formal histopathology of the appendix demonstrated acute inflammation in 2.13% of patients (*n* = 9) and suppurative inflammation in 7.33% (*n* = 31). Most patients had gangrenous appendicitis and/or perforation (*n* = 383; 90.54%).

All patients received post-operative intravenous antibiotics for an average length of 4.3 days (SD ± 1.78). One quarter of patients (*n* = 104; 24.59%) received post-operative oral antibiotics; average length of administration was 6.2 days (SD ± 2.69).

Intra-abdominal abscess (IAA) was a complication in 23 patients (5.44%). Over half of these patients were re-admitted following discharge (*n* = 13; 56.52%). The reasons for re-presentation to hospital were most commonly abdominal pain (69.23%) and abdominal pain with fever (15.38%). A small number had undergone outpatient cross-sectional imaging suggesting a post-operative abscess (15.38%). Of the 23 patients who developed post-operative IAA, 11 (47.83%) patients were diagnosed using clinical findings alone and 12 (52.17%) had an inpatient CT to aid diagnosis. Eighteen patients underwent laparoscopic washout for management. Six (*n* = 6, 38.89%) of these patients were found to have a post-operative IAA; the remaining patients were found to have a small bowel ileus (*n* = 2), small bowel obstruction (*n* = 1), RIF haematoma (*n* = 1), fibrin deposition (*n* = 2), and turbid free-fluid (*n* = 5). Only one patient had a negative post-operative laparoscopy (*n* = 1; 5.55%). Duration of symptoms pre-operatively was not found to have a significant effect on post-operative complications (see appendices).

The quantitative and categorical variables analysed that were statistically significant in at least one comparative group are summarised in Tables 2 and 3, respectively. Please see appendices for full data analysis.

**Table 2:** Analysis of significant quantitative variables across all three comparative groups

	Post-op IAA	No Post-op IAA	*p* value	Post-op IAA and Washout	No Post-op IAA or Washout	*p* value	Washout	No Washout	*p* value
Initial CRP (mean ± SD, *mg/L*) (n)	134.80 ± 115.15 (14)	95.05 ± 91.91 (253)	0.1216	**134.80 ± 134.31** (23)	**93.59 ± 88.18** (244)	**0.0430**	134.80 ± 167.50 (9)	95.32 ± 89.87 (257)	0.2128
Post-operative IV antibiotic duration (mean ± SD, *days*) (n)	**6.30 ± 3.47** (23)	**4.16 ± 1.56** (400)	**< 0.0001**	**6.34 ± 2.77** (41)	**4.06 ± 1.48** (382)	**< 0.0001**	**6.39 ± 1.58** (18)	**4.18 ± 1.73** (404)	**< 0.0001**

**Table 3:** Analysis of significant categorical variables across all three comparative groups

	Post-op IAA vs. No Post-op IAA			Post-op IAA and Washout vs. No Post-op IAA or Washout			Washout vs. No Washout		
	Odds ratio	95% CI	*p*value	Odds ratio	95% CI	*p*value	Odds ratio	95% CI	*p*value
Perforated appendix	2.85	0.98–7.86	0.0725	**4.60**	**1.77–11.04**	**0.0005**	**10.36**	**1.79 to 109.6**	**0.0046**

### Post-operative IAA vs. No Post-operative IAA

Post-operative IV antibiotic duration (*p* < 0.0001) was significantly longer in the group that developed post-operative IAA.

### Post-operative IAA and Lap Washout vs. No Post-operative IAA and No Lap Washout

Post-operative IV antibiotic duration (*p* < 0.0001) was significantly longer and initial CRP (i.e., CRP taken on admission) (*p* = 0.0430) was significantly elevated in the group that developed post-operative IAA and underwent laparoscopic washout. Appendiceal perforation (*p* = 0.0005) was significant when compared to the group that did not develop post-operative IAA or require laparoscopic washout. Histologically inflamed and gangrenous/perforated appendicitis had no impact on the two groups (see appendix 4). Clinical findings of RIF peritonism and CT free fluid also did not show significant differences between the groups (see appendix 4).

### Lap Washout vs. No Lap Washout

Post-operative IV antibiotic duration (*p* < 0.0001) was longer and appendiceal perforation (*p* = 0.0046) more common in the group that received laparoscopic washout.

## Discussion

The results of the present study are three-fold. Firstly, they identify a novel use for CRP as a biochemical marker in the context of identifying those patients at greater risk of developing post-operative IAA. Secondly, these results suggest the development of post-operative IAA and need for laparoscopic washout occurs despite prolonged use of IV antibiotics following appendicectomy. Lastly, these results highlight the complex nature of acute appendicitis particularly with respect to factors influencing the development of post-operative IAA.

The identification of CRP on admission as a pre-operative predictor for patients who are likely to develop post-operative IAA and need laparoscopic washout is of clinical significance, as this suggests a role for minimally invasive pre-operative testing in predicting clinical prognosis and directing course of management. To the best of our knowledge, CRP has not previously been implicated as a risk factor for developing post-operative IAA, however its role in other acute disease states is well characterised. For example, CRP levels are used as a criterion to rule out, with a high probability, the presence of necrosis in acute pancreatitis [13].

The results of the present study suggest that development of post-operative IAA and need for laparoscopic washout is independent of post-operative IV antibiotic duration, as those who developed post-operative IAA and received laparoscopic washout had a significantly longer course of post-operative IV antibiotics. Currently, in Australia, post-operative antibiotic use is determined by hospital-dependent policies and can depend on other factors, including the preference of the operating surgeon and presence of clear instructions for post-operative antibiotics. At our institution, patients with gangrenous or perforated appendicitis receive IV antibiotics for 5 days regardless of clinical progress, and patients with suppurative appendicitis receive IV antibiotics for 2 days, as a preventative measure against developing post-operative IAA and needing laparoscopic washout. However, our data suggests these patients developed an abscess requiring laparoscopic washout despite a longer duration of antibiotics.

Kimbrell et al. have shown previously there is no difference in the rate of developing post-operative IAA when antibiotics are ceased 24 h post-operatively as compared to patients in whom they are continued for more than 24 h [14]. A systematic review on postoperative antibiotic use in adults with acute appendicitis recommends a course of broad-spectrum IV antibiotics for 3–5 days in cases of appendiceal perforation, however no recommendation is made as to when antibiotic cessation is appropriate. [15]. Further data has shown that prolonging antibiotic therapy in both uncomplicated and complicated appendicitis does not alter the incidence of IAA, however in patients with complicated appendicitis, cessation of intravenous antibiotics in the presence of leukocytosis and fever results in an increased incidence of IAA even if oral antibiotics have been commenced [16]. In summary, while post-operative antibiotics may be of benefit, the optimal duration of treatment has yet to be clearly defined although if ceased in the presence of persistent clinical features of a systemic inflammatory response this is likely to result in an intra-abdominal abscess.

The intra-operative pathological findings (as determined by the operating surgeon) frequently determine subsequent clinical management and overall length of hospital stay. In this study, average length of hospital stay was 5.3 days. The possible discrepancy between the qualitative and histopathological findings of the appendix, which are received two weeks post-operatively, raise the possibility patients are potentially either over- or undertreated. In this study operative records were not reviewed. Future studies are underway in order to correlate intra-operative descriptors used to guide management with histopathological results in order to optimise patient care and hospital spending.

Interestingly, the clinical findings of RIF peritonism and free fluid on cross-sectional imaging did not help differentiate between patients that developed post-operative IAA and those that did not. This finding suggests there is a limitation in both clinical examination and radiological investigations when used in the post-operative setting to determine which patients have (or are developing) IAA.

Non-operative management of acute appendicitis has been discussed in the literature, particularly for management in paediatric populations. Research has shown a promising role for non-operative management in uncomplicated, early, acute appendicitis, suggesting it is feasible, safe, cost effective and more favourable among parents and patients [17, 18]. A review by Wilms et al. suggests non-operative management of uncomplicated acute appendicitis in all age groups is associated with fewer complications but may be less efficacious than surgical management [19]. In non-operative management, criteria for determining suitable patients have yet to be developed. The identification of suitable patients for non-operative management has previously relied on CT imaging which is difficult and contentious in the paediatric population, due to the risks associated with radiation exposure. Our results identify a role for CRP as a predictor of the success of non-operative management, mitigating the use of imaging. Further studies with larger patient cohorts are required to better characterise CRP levels in acute appendicitis and its potential clinical role.

We acknowledge there are several limitations to the present study. Although a retrospective analysis we thoroughly assessed for confounding variables, however, it is possible these have not been fully controlled for. We acknowledge the possibility of a small subgroup of patients that may have been lost to follow up if they have developed a complication and represented to a different hospital, however local referral patterns in our local health district would necessitate patient transfer back to our hospital for intervention or, at the very least, discussion with our hospital regarding patient management. In addition, the number of patients presenting with post-operative IAA and/or laparoscopic washout constitute only a small cohort of cases, compounded by missing data points for some patients across the groups. Our study does however cover a robust and large patient group; the selection criteria was broad to catch the entirety of the patient population undergoing laparoscopic appendicectomy for acute appendicitis during the specified time period. We acknowledge our number of complicated cases is larger than that represented in the literature. This is likely the result of our hospital covering a large geographical area servicing a low socio-economic demographic.

## Appendix 1

Analysis of quantitative variables between patient group who developed post-operative IAA compared to those who did not develop post-operative IAA

**Table:**

	Post-op IAA	No Post-op IAA	*p* value
Initial CRP (mean ± SD, mg/L) (n)	134.80 ± 115.15 (14)	95.05 ± 91.91 (253)	0.1216
Initial WCC (mean ± SD, x10^9^ cells/L) (n)	16.56 ± 3.64 (23)	15.17 ± 4.75 (398)	0.1672
Initial albumin (mean ± SD, g/dL) (n)	38.38 ± 4.73 (21)	39.48 ± 5.03 (345)	0.3279
Initial bilirubin (mean ± SD, mg/dL) (n)	16.43 ± 7.45 (21)	17.55 ± 10.18 (343)	0.6187
Initial neutrophil count (mean ± SD, x10^9^ cells/L) (n)	13.58 ± 3.92 (23)	12.73 ± 5.87 (398)	0.4961
DOS CRP (mean ± SD, mg/L) (n)	145 ± 97.19 (4)	114.1 ± 102.10 (107)	0.5531
DOS WCC (mean ± SD, x10^9^ cells/L) (n)	15.79 ± 4.06 (11)	14.80 ± 5.03 (218)	0.5206
Highest pre-op temperature (mean ± SD, °C) (n)	38.06 ± 0.73 (23)	37.76 ± 0.82 (400)	0.0864
Duration of symptoms (mean ± SD, days) (n)	2.80 ± 3.01 (23)	2.13 ± 2.07 (399)	0.3131
Time from admission to surgery (mean ± SD, hours) (n)	21.91 ± 18.10 (23)	19.29 ± 47.11 (400)	0.7906
Time from admission to antibiotics (mean ± SD, hours) (n)	6.70 ± 5.72 (23)	7.11 ± 7.31 (400)	0.7908
Post-operative IV antibiotic duration (mean ± SD, days) (n)	**6.30 ± 3.47** (23)	**4.16 ± 1.56** (400)	**< 0.0001**

*n* = sample size

## Appendix 2

Analysis of categorical variables between patient group who developed post-operative IAA compared to those who did not develop post-operative IAA

**Table:**

	Odds ratio	95% CI	*p* value
Abnormal temperature on admission	1.36	0.52–3.3	0.4667
Abnormal respiratory rate on admission	0.91	0.20–3.72	> 0.9999
Abnormal heart rate on admission	1.79	0.75–4.80	0.2793
DOS Temperature	1.53	0.58–3.72	0.4408
RIF peritonism	0.56	0.24–1.40	0.2258
CT free fluid	1.85	0.55–5.17	0.3907
Perforated appendix	2.85	0.98–7.86	0.0725
Histology: inflamed	2.23	0.19–16.16	0.3984
Histology: suppurative	0.56	0.05–3.34	> 0.9999
Histology: gangrenous and perforated	1.10	0.27–4.92	> 0.9999

## Appendix 3

Analysis of quantitative variables between patient group who developed post-operative IAA and required laparoscopic washout compared to those who did not develop post-operative IAA or require laparoscopic washout

**Table:**

	Post-op IAA and Washout	No Post-op IAA or Washout	*p* value
Initial CRP (mean ± SD, mg/L) (n)	**134.80 ± 134.31** (23)	**93.59 ± 88.18** (244)	**0.0430**
Initial WCC (mean ± SD, x10^9^ cells/L) (n)	15.16 ± 3.47 (41)	16.06 ± 4.81 (380)	0.2444
Initial albumin (mean ± SD, g/dL) (n)	38.28 ± 4.13 (39)	39.56 ± 5.09 (327)	0.1334
Initial bilirubin (mean ± SD, mg/dL) (n)	16.46 ± 11.68 (39)	17.61 ± 9.84 (325)	0.4996
Initial neutrophil count (mean ± SD, x10^9^ cells/L) (n)	13.14 ± 3.67 (41)	12.74 ± 5.97 (380)	0.6731
DOS CRP (mean ± SD, mg/L) (n)	140.30 ± 156.00 (9)	113.00 ± 96.28 (102)	0.4420
DOS WCC (mean ± SD, x10^9^ cells/L) (n)	15.26 ± 3.84 (25)	14.79 ± 5.11 (204)	0.6578
Highest pre-op temperature (mean ± SD, °C) (n)	37.97 ± 0.86 (41)	37.75 ± 0.81 (382)	0.1025
Duration of symptoms (mean ± SD, days) (n)	2.65 ± 2.51 (41)	2.12 ± 2.08 (381)	0.2010
Time from admission to surgery (mean ± SD, hours) (n)	19.00 ± 16.07 (41)	19.48 ± 48.13 (382)	0.9496
Time from admission to antibiotics (mean ± SD, hours) (n)	6.73 ± 7.11 (41)	7.12 ± 7.25 (382)	0.7422
Post-operative IV antibiotic duration (mean ± SD, days) (n)	**6.34 ± 2.77** (41)	**4.06 ± 1.48** (382)	**< 0.0001**

*n* = sample size

## Appendix 4

Analysis of categorical variables between patient group who developed post-operative IAA and required laparoscopic washout compared to those who did not develop post-operative IAA or require laparoscopic washout

**Table:**

	Odds ratio	95% CI	*p* value
Abnormal temperature on admission	1.48	0.70–2.99	0.2592
Abnormal respiratory rate on admission	1.04	0.38–2.88	0.9900
Abnormal heart rate on admission	1.36	0.70–2.58	> 0.4096
DOS Temperature	1.89	0.94–3.82	0.0773
RIF peritonism	1.02	0.49–2.14	> 0.9999
CT free fluid	2.04	0.79–5.22	0.1567
Perforated appendix	**4.60**	**1.77–11.04**	**0.0005**
Histology: inflamed	1.17	0.10–7.99	0.6042
Histology: suppurative	0.29	0.03–1.83	0.3420
Histology: gangrenous and perforated	2.15	0.58–9.38	0.4052

## Appendix 5

Analysis of quantitative variables between patient group who required laparoscopic washout compared to those who did not require laparoscopic washout

**Table:**

	Washout	No Washout	*p* value
Initial CRP (mean ± SD, mg/L) (n)	134.80 ± 167.50 (9)	95.32 ± 89.87 (257)	0.2128
Initial WCC (mean ± SD, x10^9^ cells/L) (n)	15.41 ± 3.23 (18)	15.22 ± 4.76 (402)	0.8688
Initial albumin (mean ± SD, g/dL) (n)	38.17 ± 3.43 (18)	39.49 ± 5.07 (348)	0.2767
Initial bilirubin (mean ± SD, mg/dL) (n)	16.50 ± 15.48 (18)	17.54 ± 9.71 (346)	0.6688
Initial neutrophil count (mean ± SD, x10^9^ cells/L) (n)	12.58 ± 3.34 (18)	12.78 ± 5.88 (402)	0.8886
DOS CRP (mean ± SD, mg/L) (n)	136.60 ± 203.83 (5)	113.10 ± 95.87 (105)	0.6162
DOS WCC (mean ± SD, x10^9^ cells/L) (n)	14.85 ± 3.76 (14)	14.82 ± 5.06 (214)	0.9835
Highest pre-op temperature (mean ± SD, °C) (n)	37.86 ± 1.01 (18)	37.77 ± 0.80 (404)	0.6259
Duration of symptoms (mean ± SD, days) (n)	2.46 ± 1.73 (18)	2.16 ± 2.15 (403)	0.4822
Time from admission to surgery (mean ± SD, hours) (n)	15.28 ± 12.50 (18)	19.65 ± 46.99 (404)	0.6939
Time from admission to antibiotics (mean ± SD, hours) (n)	6.78 ± 8.76 (18)	7.11 ± 7.17 (404)	0.8507
Post-operative IV antibiotic duration (mean ± SD, days) (n)	**6.39 ± 1.58** (18)	**4.18 ± 1.73** (404)	**< 0.0001**

*n* = sample size

## Appendix 6

Analysis of categorical variables between patient group who required laparoscopic washout compared to those who did not require laparoscopic washout

**Table:**

	Odds ratio	95% CI	*p* value
Female (vs. male)	0.52	0.20–1.40	0.2344
Abnormal temperature on admission	1.58	0.59–4.16	0.4018
Abnormal respiratory rate on admission	1.20	0.26–4.64	0.6841
Abnormal heart rate on admission	0.95	0.36–2.41	> 0.9999
DOS Temperature	2.29	0.88–5.99	0.1433
RIF peritonism	3.09	0.74–13.75	0.1742
CT free fluid	2.28	0.51–12.15	0.4251
Perforated appendix	**10.36**	**1.79 to 109.6**	**0.0046**
Histology: inflamed	0.89	0.05–15.86	> 0.9999
Histology: suppurative	0.32	0.019–5.44	0.3837
Histology: gangrenous and perforated	4.11	0.24–69.5	0.3979
